# FootprintCharter: unsupervised detection and quantification of footprints in single molecule footprinting data

**DOI:** 10.1093/bioinformatics/btaf502

**Published:** 2025-09-17

**Authors:** Guido Barzaghi, Arnaud R Krebs, Judith B Zaugg

**Affiliations:** Genome Biology Unit, EMBL Heidelberg, Meyerhofstraße 1, Heidelberg, 69117, Germany; Faculty of Biosciences, Collaboration for Joint PhD Degree between EMBL and Heidelberg University, Im Neuenheimer Feld 234, Heidelberg, 69120, Germany; Genome Biology Unit, EMBL Heidelberg, Meyerhofstraße 1, Heidelberg, 69117, Germany; Structural and Computational Biology Unit, EMBL Heidelberg, Meyerhofstraße 1, Heidelberg, 69117, Germany; Department of Biomedicine, University of Basel, Hebelstrasse 20, Basel, 4031, Switzerland

## Abstract

**Summary:**

Single molecule footprinting profiles the heterogeneity of TF occupancy at *cis*-regulatory elements across cell populations at unprecedented resolution. The single molecule nature of the data in principle allows for observing the footprint of individual transcription factors and nucleosomes. However, we currently lack algorithms to quantify these occupancy patterns of chromatin binding factors in an automated way and without prior assumptions on their genomic location. Here we present *FootprintCharter*, an unsupervised tool to detect and quantify footprints for transcription factors (TFs) and nucleosomes from single molecule footprinting data. After detection, TF footprints can be labeled with orthogonal motif annotations provided by the user. *FootprintCharter* allows for the quantification of complex molecular states such as positioning of unphased nucleosomes and combinatorial co-binding of multiple TFs.

**Availability and implementation:**

*FootprintCharter* is freely available on Bioconductor with version 2.2.0 of https://bioconductor.org/packages/SingleMoleculeFootprinting through the functions *FootprintCharter*, *PlotFootprints*, and *Plot_FootprintCharter_SM*.

## 1 Introduction

**Figure 1.**
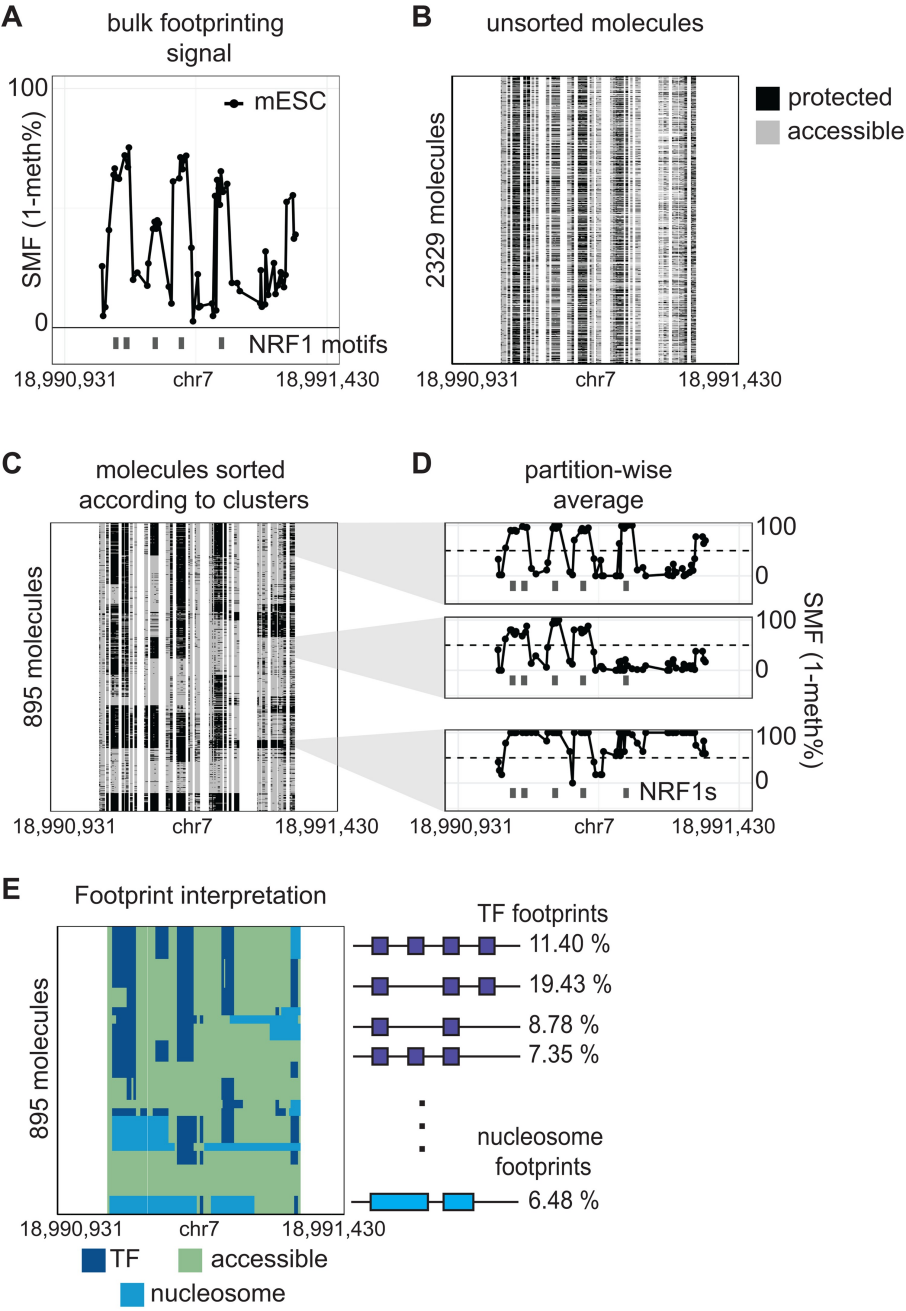
*FootprintCharter* allows for the unsupervised quantification of single molecule footprinting patterns at complex loci. (A) Example locus bound by NRF1 at five distinct motifs. The *y*-axis shows the average SMF signal (1-methylation %) at individual GpCs and CpGs, which can be interpreted as a measure of genomic occupancy frequency. Already the average SMF signal reveals short, but highly frequent, footprints overlapping the TF motifs. (B) Heatmap displaying the unsorted molecules covering the locus. Each column shows an individual genomic cytosine and each row a single molecule. Each single cytosine on a single molecule, is colored according to its binary occupancy status (accessible in grey, protected in black). (C) Single molecules sorted according to the unsupervised clustering results. (D) Cluster-wise average SMF signal used for footprint detection. Short footprints (5–75 bp) are interpreted as TFs, footprints wider than 120 bp are interpreted as nucleosomes. (E) Single molecules annotated with biological state by footprint detection results (TF in purple, accessible in green, nucleosome in blue).

Single molecule footprinting (SMF) maps DNA–protein interactions at *cis*-regulatory elements at single molecule resolution. It does so by marking cytosines that are not protected by DNA-bound proteins with exogenous methyl-transferases and by measuring those methylation marks with bisulfite sequencing (Krebs *et al.* 2017, Kleinendorst *et al.* 2021). Recent studies demonstrated the ability of SMF to measure the heterogeneity of transcription factor (TF) binding (Sönmezer *et al.* 2021) (Fig. 1A), polymerase occupancy (Krebs *et al.* 2017, Chatsirisupachai *et al.* 2025) and chromatin accessibility (Kreibich *et al.* 2023, Baderna *et al.* 2025) at the single molecule level across cell populations. Yet, we are still lacking a tool for the automatic quantification of occupancy patterns of chromatin binding factors without prior assumptions on their genomic location such as mapped TF motifs or transcription start sites.

Complementing the repertoire of computational tools for SMF (Kleinendorst *et al.* 2021, Doughty *et al.* 2024) and of unsupervised tools for single molecule genomics (Tullius *et al.* 2024, Vollger *et al.* 2024), we developed *FootprintCharter*, a novel unsupervised molecular classifier and footprint detection algorithm tailored for SMF. Both our previous single molecule sorting method (Kleinendorst *et al.* 2021) as well as the single-molecule state calling model reported by Doughty *et al.* require prior enumeration of all the footprinting patterns expected for a genomic locus. Instead, *FootprintCharter* explicitly detects footprints for transcription factors (TFs) and nucleosomes and quantifies their frequency across cell populations. Notably, this tool performs these quantifications independently of orthogonal TF motif annotations. Instead, those annotations can be used downstream to label the discovered TF footprints. *FootprintCharter* allows for the estimation of frequencies for complex molecular patterns along single molecules, including various combinatorics of large TF clusters and nucleosome arrays. The *FootprintCharter* implementation is freely available as part of our previously reported *SingleMoleculeFootprinting* R/Bioconductor package (Gentleman *et al.* 2004, Huber *et al.* 2015, Kleinendorst *et al.* 2021).

## 2 Results

### 2.1 Input

*FootprintCharter* takes as input single molecule methylation matrixes as produced by the function *CallContextMethylation* of our *SingleMoleculeFootprinting* R/Bioconductor package (Fig. 1B). Internally, methylation calling at the single molecule level is carried using the Bioconductor package *QuasR* (Gaidatzis *et al.* 2015). The *QuasR* output is converted to sparse matrixes using the *Matrix* R package (Bates *et al.* 2000).

*FootprintCharter* quantifies footprints independently of prior TF motif annotations. Therefore, it focuses on user-defined genomic intervals encoded as *GRanges* objects from the *GenomicRanges* R/Bioconductor package (Lawrence *et al.* 2013). Read length should be considered when defining these windows. For instance, we experienced that 80bps is a suitable interval width for 150 paired-end SMF data.

### 2.2 Unsupervised clustering of molecules

First, binary methylation values are smoothed along single molecules by computing, at each genomic position, a rolling mean over a 40 bp window. Secondly, a matrix of pairwise Euclidean distances among single molecules is computed using the cytosine methylation signal along molecules as features. This matrix is computed with the *parDist* function of the *parallelDist* R package. Unsupervised clustering is performed on the resulting matrix using the partitioning around medoids algorithm (pam), implemented in the *pam* function of the *cluster* R package (Schubert and Rousseeuw 2021) (Fig. 1C). The number of clusters (*k*) is initially set by the user and iteratively reduced by *FootprintCharter* until all clusters are populated by at least *n* molecules, where *n* is also user-defined. By capping the number of computed clusters, the parameter *n* aids the grouping of molecules into recurring footprinting patterns and further reduces the impact of technical noise (Fig. 1A and B, available as supplementary data at *Bioinformatics* online). We set default values at *k *= 16 and *n *= 5 based on empirical evidence from analyzing SMF datasets produced with a bait-capture enrichment step (Kleinendorst *et al.* 2021, Sönmezer *et al.* 2021).

### 2.3 Footprint detection

For each cluster, which corresponds to a biologically distinct chromatin occupancy pattern, *FootprintCharter* computes the average SMF signal at each cytosine using the input binary methylation values. Within those average tracks, cytosines are defined as part of footprints if their resulting median SMF signal exceeds 50% (Fig. 1D). Consecutive stretches of footprinted cytosines that are between 5 and 75 bp in width are interpreted as TF footprints. Stretches longer than 120 bp are interpreted as nucleosome footprints. Footprints are considered as “unrecognized” if they are not flanked by accessible cytosines on both sides since their full width cannot be established, as it happens at the edge of molecules. Furthermore, the tool considers stretches shorter than 5 bp to be artifacts and labels them as “noise” (Fig. 1E).

### 2.4 TF footprint aggregation and annotation

*FootprintCharter* detects footprints separately for each cluster. However, measuring TF-DNA interactions is associated with a certain level of biological and technical noise. We for instance observed that the same protein-DNA contact might result in footprints that are partially shifted along different molecules. To account for this when estimating the frequency of footprints across cells, we aggregate TF footprints when they overlap by at least 75% of their width.

Users can also provide TF motif annotations, such as those from the JASPAR database (Mathelier *et al.* 2016), to label TF footprints. If provided, this annotation will also be used to aggregate TF footprints.

### 2.5 Output and data visualization

The steps above are performed by the *FootprintCharter* function. The output is a data frame reporting the coordinates and frequencies of the TF and nucleosome footprints detected around the input genomic interval.

The data transformations performed by *FootprintCharter* can be visualized using the functions *PlotFootprints* and *Plot_FootprintCharter_SM*, as exemplified in Fig. 1D and E respectively. Both functions return *ggplot2* objects which can be manipulated and customized with standard geom_* grammar.

### 2.6 Note on generalization and future developments

Albeit specific to SMF, *FootprintCharter* does not make assumptions on the footprinted context and it could work with data that include adenosine methylation such as Fiber-seq (Stergachis *et al.* 2020) or SMAC-seq (Shipony *et al.* 2020). However, because the running time of the initial smoothing step grows linearly with the molecule length, further tool development might be necessary to exclude this step. For guidance, we include a runtime and memory footprint analysis (Fig. 1C and D, available as supplementary data at *Bioinformatics* online).

*FootprintCharter* is intended as an unsupervised algorithm, but it still relies on orthogonal motif annotations to label TF footprints. A further development could include the *de novo* discovery of motifs from TF footprints aimed at labeling the orphan footprints discovered by the tool.

## Supplementary Material

btaf502_Supplementary_Data

## Data Availability

The data used in this study was obtained from (Sönmezer *et al.* 2021) under ArrayExpress accession number E-MTAB-9033. The code used to generate the figure is available at https://github.com/Krebslabrep/Barzaghi-et-al_application-note.git and archived on Zenodo (DOI: https://doi.org/10.5281/zenodo.16896124). More details on SMF data pre-processing can be found in our previously published protocol (Kleinendorst *et al.* 2021).

## References

[btaf502-B1] Baderna V , Barzaghi G, Kleinendorst R et al Cumulative TF binding and H3K27 Acetylation drive enhancer activation frequency. biorxiv, 2025. 10.1101/2025.03.26.645413 PMC13447113 42538372

[btaf502-B2] Bates D , Maechler M, Jagan M. Matrix: sparse and dense matrix classes and methods. 2000. 10.32614/CRAN.package.Matrix

[btaf502-B3] Chatsirisupachai K , Moene CJI, Kleinendorst R et al Mouse promoters are characterised by low occupancy and high turnover of RNA polymerase II. Mol Syst Biol. 2025;21:447–71. 10.1038/s44320-025-00094-5 PMC12048509 40164797

[btaf502-B4] Doughty BR , Hinks MM, Schaepe JM et al Single-molecule states link transcription factor binding to gene expression. Nature 2024;636:745–54. http://doi/10.1038/s41586-024-08219-w 10.1038/s41586-024-08219-w PMC12326879 39567683

[btaf502-B5] Gaidatzis D , Lerch A, Hahne F et al QuasR: quantification and annotation of short reads in R. Bioinformatics 2015;31:1130–2. 10.1093/bioinformatics/btu781 25417205 PMC4382904

[btaf502-B6] Gentleman RC , Carey VJ, Bates DM et al Bioconductor: open software development for computational biology and bioinformatics. Genome Biol 2004;5:R80. 10.1186/gb-2004-5-10-r80 15461798 PMC545600

[btaf502-B7] Huber W , Carey VJ, Gentleman R et al Orchestrating high-throughput genomic analysis with Bioconductor. Nat Methods 2015;12:115–21. 10.1038/nmeth.3252 25633503 PMC4509590

[btaf502-B8] Kleinendorst RWD , Barzaghi G, Smith M et al Genome-wide quantification of transcription factor binding at single-DNA-molecule resolution using methyl-transferase footprinting. Nat Protoc 2021;16:5673–706. 10.1038/s41596-021-00630-1 34773120 PMC7613001

[btaf502-B9] Krebs AR , Imanci D, Hoerner L et al Genome-wide single-molecule footprinting reveals high RNA polymerase II turnover at paused promoters. Mol Cell 2017;67:411–22.e4. 10.1016/j.molcel.2017.06.027 28735898 PMC5548954

[btaf502-B10] Kreibich E , Kleinendorst R, Barzaghi G et al Single-molecule footprinting identifies context-dependent regulation of enhancers by DNA methylation. Mol Cell 2023;83:787–802.e9. 10.1016/j.molcel.2023.01.017 36758546

[btaf502-B11] Lawrence M , Huber W, Pagès H et al Genomic ranges’, (A. Prlic, Ed.). PLoS Comput Biol 2013;9:e1003118. 10.1371/journal.pcbi.1003118 23950696 PMC3738458

[btaf502-B12] Mathelier A , Fornes O, Arenillas DJ et al JASPAR 2016: a major expansion and update of the open-access database of transcription factor binding profiles. Nucleic Acids Res 2016;44:D110–5. 10.1093/nar/gkv1176 26531826 PMC4702842

[btaf502-B13] Schubert E , Rousseeuw PJ. Fast and eager k -medoids clustering: O (k) runtime improvement of the PAM, CLARA, and CLARANS algorithms. Inf Syst 2021;101:101804. 10.1016/j.is.2021.101804

[btaf502-B14] Shipony Z , Marinov GK, Swaffer MP et al Long-range single-molecule mapping of chromatin accessibility in eukaryotes. Nat Methods 2020;17:319–27. 10.1038/s41592-019-0730-2 32042188 PMC7968351

[btaf502-B15] Sönmezer C , Kleinendorst R, Imanci D et al Molecular co-occupancy identifies transcription factor binding cooperativity in vivo. Mol Cell 2021;81:255–67.e6. 10.1016/j.molcel.2020.11.015 33290745 PMC7612519

[btaf502-B16] Stergachis AB , Debo BM, Haugen E et al Single-molecule regulatory architectures captured by chromatin fiber sequencing. Science 2020;368:1449–54. 10.1126/science.aaz1646 32587015

[btaf502-B17] Tullius TW , Isaac RS, Dubocanin D et al RNA polymerases reshape chromatin architecture and couple transcription on individual fibers. Mol Cell 2024;84:3209–22.e5. 10.1016/j.molcel.2024.08.013 39191261 PMC11500009

[btaf502-B18] Vollger MR , Swanson EG, Neph SJ et al A haplotype-resolved view of human gene regulation. biorxiv, 2024. 10.1101/2024.06.14.599122

