# Supplementary material for: FootprintCharter: unsupervised detection and quantification of footprints in single molecule footprinting data
Source: Bioinformatics. 2025 Sep 17;41(10):btaf502. doi: 10.1093/bioinformatics/btaf502 (PMC12490827; doi:10.1093/bioinformatics/btaf502)
Supplement: btaf502_Supplementary_Data [file btaf502_supplementary_data.pdf]

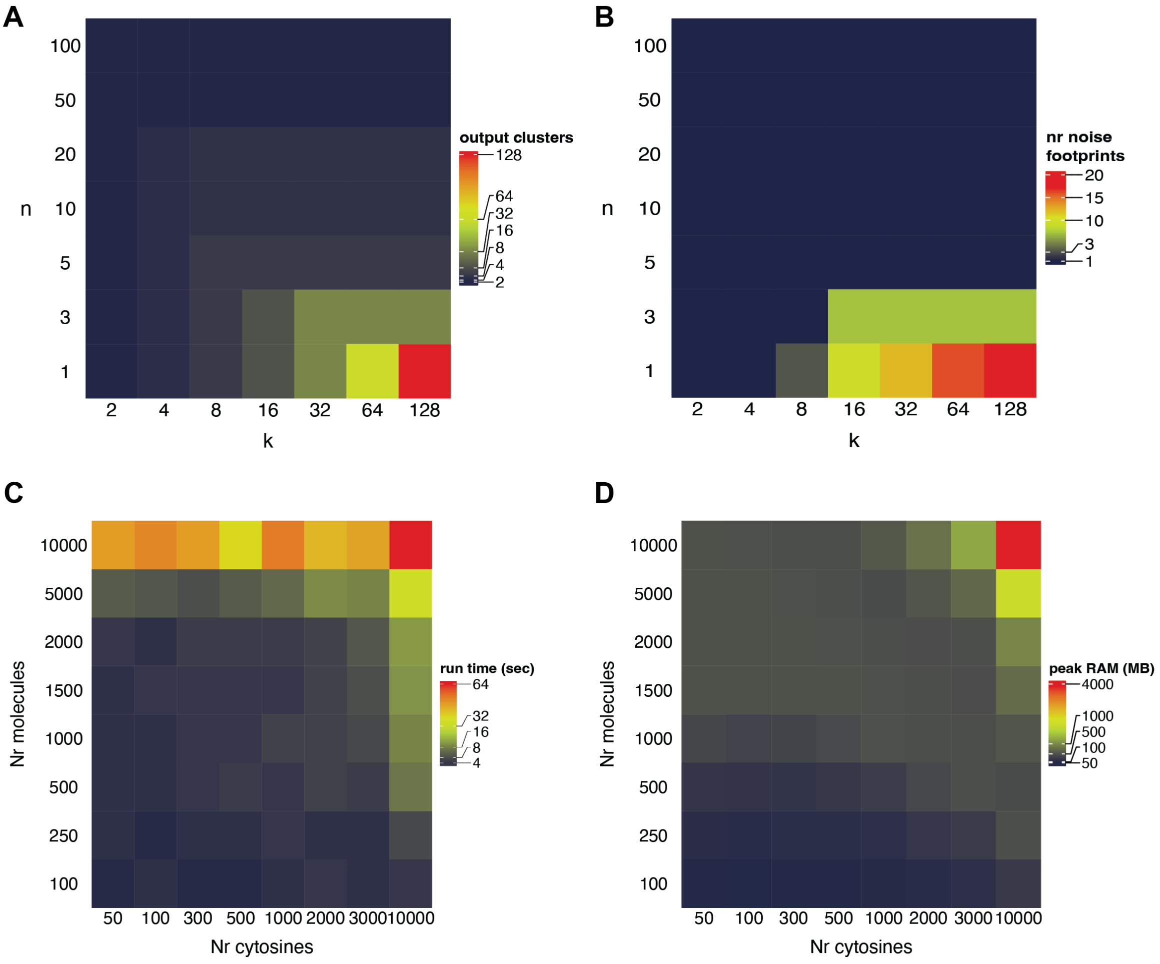

Supplementary Figure 1. *FootprintCharter* benchmarks. (A) Number of single molecule clusters computed by *FootprintCharter* as a function of varying values for parameters *k* and *n*. The parameter *n* caps the number of computed clusters by imposing the minimal number of molecules per cluster. With *n*=1, the number of computed clusters equals *k*. When *n* >= 5 the number of clusters remains fairly stable regardless of the initial value of *k*. (B) Number of "noise" footprints (footprint stretches <5pb) detected as a function of *k* and *n*. Increasing *n* reduces the number of "noise" footprints which result by increasing *k*. The number of "noise" footprints remains stable when *n* is equal or larger than 5, motivating the default value (C) Run time in seconds of *FootprintCharter* increases as a function of the number of molecules and cytosines in the input matrix. (D) Peak RAM footprint of *FootprintCharter* increases as a function of the number of molecules and cytosines in the input matrix.
